# Supplementary material for: FERN – a Java framework for stochastic simulation and evaluation of reaction networks
Source: BMC Bioinformatics. 2008 Aug 29;9:356. doi: 10.1186/1471-2105-9-356 (PMC2553347; doi:10.1186/1471-2105-9-356)
Supplement: Additional file 1 — FERN distribution, Version 1.3. This archive contains the FERN source code and binaries as well as documentation and example models in FernML and SBML. [file 1471-2105-9-356-S1.zip › fern/doc/javadoc/index-files/index-12.html]

M-Index


---


|  |  |  |
| --- | --- | --- |
| **Overview** \| Package \| Class \| Use \| **Tree** \| **Deprecated** \| **Index** \| **Help** | |  |
| **PREV LETTER**   **NEXT LETTER** | **FRAMES**    **NO FRAMES**     **All Classes** |  |


A B C D E F G H I J L M N O P R S T U V 

---


## **M**

**main(String[])** - Static method in class fern.benchmark.RandomNumber: **main(String[])** - Static method in class fern.benchmark.SimulatorCorrectness: **main(String[])** - Static method in class fern.cellDesigner.ui.MainFrame: **main(String[])** - Static method in class fern.cytoscape.ui.MainFrame: **main(String[])** - Static method in class fern.example.AutocatalyticNetworkExample: **main(String[])** - Static method in class fern.example.DecayingDimerizingHistogramDistances: **main(String[])** - Static method in class fern.example.DecayingDimerizingInteractive: **main(String[])** - Static method in class fern.example.DecayingDimerizingPlots: **Deprecated.** **main(String[])** - Static method in class fern.example.Dsmts: **main(String[])** - Static method in class fern.example.IrreversibleIsomerization: **main(String[])** - Static method in class fern.example.LacYComplete: **main(String[])** - Static method in class fern.example.LacYHistogramDistances: **main(String[])** - Static method in class fern.example.LacZ: **main(String[])** - Static method in class fern.example.MapkBenchmark: **main(String[])** - Static method in class fern.example.MichaelisMentenKinetic: **main(String[])** - Static method in class fern.example.SBMLMathTreeTest: Dumb the MathTrees of an SBML network to Stdout **main(String[])** - Static method in class fern.Start: **MainFrame** - Class in fern.cellDesigner.ui: **MainFrame(FernCellDesignerPlugin)** - Constructor for class fern.cellDesigner.ui.MainFrame: **MainFrame** - Class in fern.cytoscape.ui: **MainFrame(FernVisualStyle)** - Constructor for class fern.cytoscape.ui.MainFrame: **MapkBenchmark** - Class in fern.example: Use the signal transduction pathway network of the epidermal growth factor proposed by [1] to introduce the benchmark system. **MapkBenchmark()** - Constructor for class fern.example.MapkBenchmark: **MathTree** - Class in fern.network.sbml: Representation of am evaluation tree. **MathTree(Network, ASTNode, Map<String, Double>, Map<String, Double>, Map<String, Integer>)** - Constructor for class fern.network.sbml.MathTree: Creates a MathTree from an libsbml `ASTNode`. **MathTree.ConstLeaf** - Class in fern.network.sbml: **MathTree.ConstLeaf(double)** - Constructor for class fern.network.sbml.MathTree.ConstLeaf: **MathTree.GlobalLeaf** - Class in fern.network.sbml: **MathTree.GlobalLeaf(String)** - Constructor for class fern.network.sbml.MathTree.GlobalLeaf: **MathTree.InnerNode** - Class in fern.network.sbml: **MathTree.InnerNode(MathTree.Node[], int)** - Constructor for class fern.network.sbml.MathTree.InnerNode: **MathTree.Node** - Class in fern.network.sbml: **MathTree.Node()** - Constructor for class fern.network.sbml.MathTree.Node: **MathTree.VarLeaf** - Class in fern.network.sbml: **MathTree.VarLeaf(String, int)** - Constructor for class fern.network.sbml.MathTree.VarLeaf: **max(Iterable<Integer>)** - Static method in class fern.tools.NumberTools: Gets the maximal value within the given `Iterable`. **merge(GnuPlot)** - Method in class fern.tools.gnuplot.GnuPlot: Merges another gnuplot object with this one by add its axes and copy its default style to the axes (if they have no style). **MichaelisMentenKinetic** - Class in fern.example: The most basic example uses the famous enzyme kinetics equation by Michaelis and Menten S + E <-> ES -> P to introduce fundamental loading and repeated simulation of reaction networks. **MichaelisMentenKinetic()** - Constructor for class fern.example.MichaelisMentenKinetic: **min(Iterable<Integer>)** - Static method in class fern.tools.NumberTools: Gets the minimal value within the given `Iterable`. **model** - Variable in class fern.network.sbml.SBMLNetwork: The sbml model created by libsbml. **modelClosed(PluginSBase)** - Method in class fern.cellDesigner.FernCellDesignerPlugin: **modelOpened(PluginSBase)** - Method in class fern.cellDesigner.FernCellDesignerPlugin: **modelSelectChanged(PluginSBase)** - Method in class fern.cellDesigner.FernCellDesignerPlugin: **ModifierNetwork** - Class in fern.network.modification: Base class for modified networks, which implements the full `Network` interface. **ModifierNetwork(Network)** - Constructor for class fern.network.modification.ModifierNetwork: Creates a `ModifierNetwork` from an original network. **mu** - Variable in class fern.simulation.algorithm.AbstractTauLeapingPropensityBoundSimulator: **MultiAmountIntervalObserver** - Class in fern.simulation.observer: Observes amounts of molecule species repeatedly after certain intervals. **MultiAmountIntervalObserver(Simulator, String, double, int...)** - Constructor for class fern.simulation.observer.MultiAmountIntervalObserver: Creates the observer for a given simulator, a given interval and given species indices **MultiAmountIntervalObserver(Simulator, String, double, String...)** - Constructor for class fern.simulation.observer.MultiAmountIntervalObserver: Creates the observer for a given simulator, a given interval and given species names.

---


|  |  |  |
| --- | --- | --- |
| **Overview** \| Package \| Class \| Use \| **Tree** \| **Deprecated** \| **Index** \| **Help** | |  |
| **PREV LETTER**   **NEXT LETTER** | **FRAMES**    **NO FRAMES**     **All Classes** |  |


A B C D E F G H I J L M N O P R S T U V 

---
